# Case Report: Isolate Congenital Coronary Artery Fistula With Giant Artery Aneurysm in a Neonate

**DOI:** 10.3389/fcvm.2021.633840

**Published:** 2021-06-16

**Authors:** Haoyong Yuan, Zhongshi Wu, Qin Wu, Ting Lu, Yilun Tang, Can Huang

**Affiliations:** ^1^Department of Cardiovascular Surgery, The Second Xiangya Hospital, Central South University, Changsha, China; ^2^Engineering Laboratory of Hunan Province for Cardiovascular Biomaterials, Changsha, China

**Keywords:** neonate, congenital heart disease, coronary artery fistula, coronary artery aneurysm, cardiovascular surgery

## Abstract

A rare case of neonatal congenital coronary artery, right ventricle fistula with giant coronary artery aneurysm formation, was reported. Computed tomography angiography demonstrated the dilated and tortuous tunnel arising from the right aortic sinus and traversing the epicardial surface before opening into the anterolateral aspect of the RV. Successful surgical repair was performed with a patch closure of the fistula and coronary angioplasty. The postoperative recovery was uneventful. Our experience of this rare congenital heart disease demonstrated that early surgical repair of coronary artery fistula and coronary angioplasty in the neonate can be performed safely. Further study is needed to seek the basis on this.

## Introduction

Congenital coronary artery fistula (CAF) is a communication between a coronary artery and a cardiac chamber, with an incidence of 0.2% of all congenital heart disease (1). Most CAFs originate from the right coronary artery (RCA) and drain into the right heart chamber. Coronary artery dilation over the diameter of a normal adjacent segment by 1.5 times is defined as coronary artery aneurysm (CAA), and an aneurysm over 8 mm in diameter is termed as a giant CAA in children according to the AHA classification (2). CAF associated with aneurysms is extremely rare in neonate patients. Symptoms range from spontaneous closure to a life-threatening condition, depending on the size, and exit point of the fistula. Giant CAA may develop sudden heart and/or respiratory failure soon after birth (2). Herein, we present a neonate diagnosed as isolate CAF with giant CAA and received successful surgery.

## Case Report

**Figure 1:**
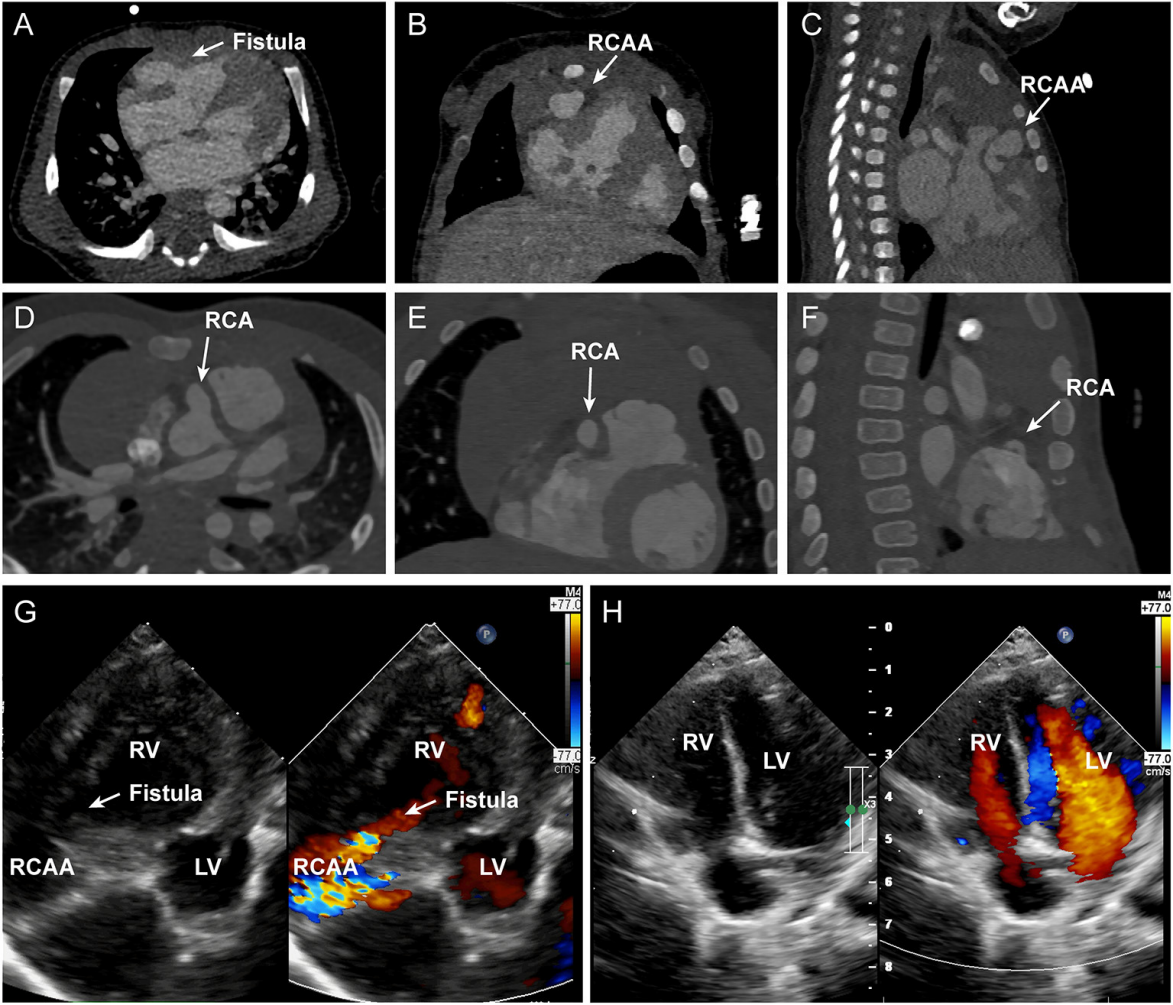
**(A–C)** depict the axial, coronal, and sagittal panel of the preoperative CTA, respectively. The white arrows indicated the fistula and RCAA. **(D–F)** depict the axial, coronal, and sagittal panel of the 6-months follow-up CTA, respectively. The white arrows indicate the mild dilated RCA. **(G,H)** show the preoperative and postoperative echocardiography images of the patient. A large fistula and RCCA to RV shunt was indicated by the arrow. RCA, right coronary artery; RCAA, right coronary artery aneurysm; LV, left ventricle; RV, right ventricle.

**Figure 2:**
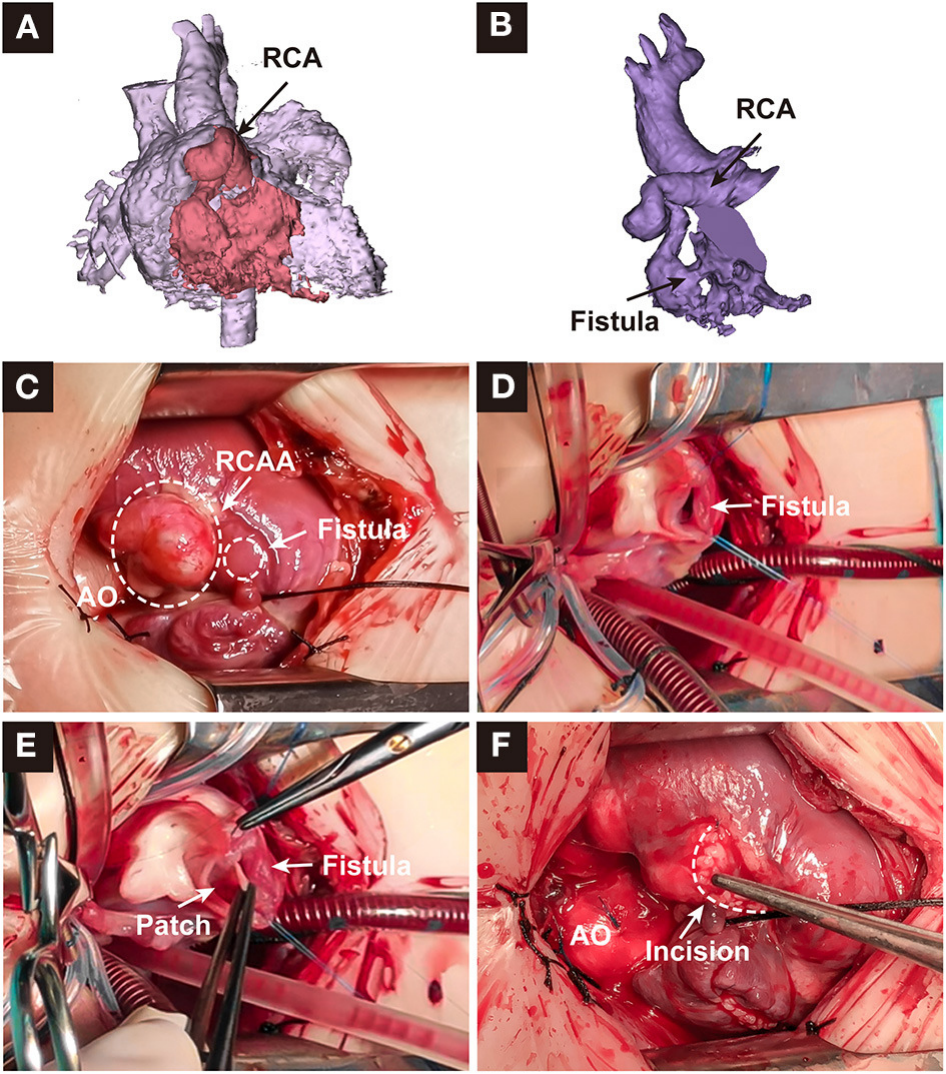
**(A,B)** depicted the reconstructed image of the heart and dilated right coronary artery. **(C–F)** depicted the surgical repair process. RCA, right coronary artery; RCAA, right coronary artery aneurysm; AO, aorta.

A 15-day-old male neonate presented with a murmur, tachypnea, and increased congestive heart failure since birth. There were no antenatal issues. Chest radiograph demonstrated cardiomegaly and pulmonary plethora, and an electrocardiogram showed left ventricular hypertrophy with strain. Transthoracic echocardiogram showed a large fistulous tunnel arising from the RCA, coursing anteriorly into a large aneurysm before entering the RV (Figure 1G). Computed tomography angiography demonstrated the dilated and tortuous tunnel arising from the right aortic sinus and traversing the epicardial surface before opening into the anterolateral aspect of the RV (Figures 1A–C, 2A,B). The left coronary system was normal. Surgical closure of the fistula was performed via median sternotomy with cardiopulmonary bypass. A huge coronary artery aneurysm appeared on the epicardial surface (Figure 2C) The dilated RCA was cut open and a true intramyocardial tunnel covered with endocardium was revealed (Figure 2D). A discrete neck of the fistula opening into the enlarged RCA was identified and closed with a bovine pericardium patch, and the RCA wall was trimmed and reconstructed with a running suture (Figures 2E,F). Antiplatelet therapy was performed by giving 3 mg/kg aspirin once a day. The postoperative recovery was uneventful. Postoperative echocardiography showed that the fistula was closed and the coronary artery aneurysm was reduced (Figure 1H). Preoperative and postoperative echocardiography data are shown in Table 1. After 6 months follow-up, the patient was in good condition without tachypnea, heart failure, and stethalgia. Six-month CTA showed that RCA was unobstructed without any thrombosis, and we just found a mild dilation of the initial part of RCA (Figures 1D–F).

**Table 1:** Echocardiography parameters before and after surgical repair.

**Parameters**	**Preoperative data**	**1-week post operation**	**6-months follow-up**
AO/PA, mm	12/16	11/11	14/16
Left atrium, mm	14	12	13
Left ventricle, mm	23	21	25
Right atrium, mm	19	17	19
Right ventricle, mm	19	17	19
LCA diameter, mm	2.1	2.2	2.5
RCA diameter, mm	6.8	6.4	6.0
Fistula diameter, mm	6.0	/	/
RCAA diameter, mm	14*11	/	/
MPAP, mmHg	41	28	28

*The echocardiography parameters of the patient before and after surgical repair. AO/PA, aorta/pulmonary artery. LCA, left coronary artery. RCA, right coronary artery. RCAA, right coronary artery aneurysm. MPAP, mean pulmonary artery pressure*.

## Discussion

In this study, we describe a neonate who developed early symptoms of congestive heart failure owing to a large congenital CAF with a coronary aneurysm. The 6-months follow-up was wonderful, which indicated that early surgical repair of CAF in the neonate can be performed safely and received good midterm results.

The obvious dilation and RCA aneurysm resulted from the 6.0 mm large fistula, which led to the preoperative symptoms. The patch repair of the fistula and the reconstruction of RCA and CCA resulted in a relatively normal RCA direction and inner diameter, which were beneficial to prevent turbulence and thrombosis, combined with the usage of aspirin. Successful surgical closure of a CAF was first reported by Bjork and Crafoord in 1947. After then, intraluminal suture closure of the CAF was reported by Symbas in 1962 and transcatheter technique was reported by Reidy in 1983 (3). The worldwide experience with surgical repair of CAF in children is limited. Surgical management is recommended to prevent myocardial infarction, coronary thrombosis, endocarditis, aneurysm formation, and fistula rupture. It is a consensus that surgical treatment should be implemented for symptomatic patients, regardless of their age. However, the timing and indication of surgery or transcatheter therapy are still controversial for asymptomatic patients. Because of the low complication rate and nearly 0% mortality, surgical repair is recommended as soon as possible to minimize the development of symptoms or long-term sequelae in the isolate CAF patients (4). At present, according to the size and anatomic course of the fistula, transcatheter device closure of the fistula has been described as an alternative to surgical closure. However, there is no evidence that transcatheter closure is safer and more effective than conventional surgical repair, especially in patients with giant CAA. Considering the heart failure and progressive dyspnea in the patient and no experience of transcatheter for neonate in our hospital, surgical repair was employed and achieved success.

Several studies reported acute and late-onset thrombosis with and without myocardial infarction (MI) after the closure of CAFs, due to a large residual vascular structure with sluggish flow angiographically (5). There are currently limited and incomplete data regarding the role of anticoagulant therapy in patients with CAF, and it is yet to determine the necessity of anticoagulant therapy after the operation. Wortham proposed that warfarin seems workable for patients with CAA. Okubo reported the postoperative use of aspirin in small dosage after the application of Amplatzer to close the CAF (6). In our patient, a dilated RCA with a giant coronary aneurysm was found. We performed coronary angioplasty to eliminate the possibility of coronary artery thrombosis after the surgery, although the coronary angioplasty that could induce death was argued to be abandoned. CTA showed a mild dilation of RCA after surgery, and 3 mg/kg aspirin was used as an antiplatelet agent. With the limited information, we recommended that coronary angioplasty should be performed and an over 6-months anticoagulant therapy should be taken for patients with a significantly dilated residual segment.

The main limitation of this study is the lack of enough cases. We cannot provide a controlled clinical research to demonstrate the availability and efficacy of our strategy. Furthermore, a longer follow-up result should be performed to observe long-term outcome.

In conclusion, we describe a neonate who developed early symptoms of congestive heart failure owing to a large congenital CAF with a giant coronary aneurysm. Early surgical repair of CAF and coronary angioplasty in the neonate can be performed safely. Multicenter longitudinal studies with larger patient numbers and more detailed anatomic and postoperative functional coronary artery evaluations are needed to better understand the best management approach and long-term outcome of this rare and intriguing disorder.

## Data Availability Statement

The original contributions generated for the study are included in the article/Supplementary Material, further inquiries can be directed to the corresponding author/s.

## Ethics Statement

Written informed consent was obtained from the individual(s), and minor(s)' legal guardian/next of kin, for the publication of any potentially identifiable images or data included in this article.

## Author Contributions

ZW and CH provide the idea and concept of this study and were in charge of study management. HY was responsible for data collection, analysis, and writing. QW contributed to echocardiography examination and provided the images. TL and YT helped to collect patients' data and were responsible for editing. All authors contributed to the article and approved the submitted version.

## Conflict of Interest

The authors declare that the research was conducted in the absence of any commercial or financial relationships that could be construed as a potential conflict of interest.

## Abbreviations

CAF: Coronary artery fistula
CAA: Coronary artery aneurysm
RCA: Right coronary artery.
